# Burst abdomen following ventriculoperitoneal shunt placement

**DOI:** 10.4103/1817-1745.66668

**Published:** 2010

**Authors:** Patrick O. Eghwrudjakpor, Iseseoma Gbobo

**Affiliations:** Department of Surgery, University of Port Harcourt Teaching Hospital, Port Harcourt, Nigeria

Sir,

Ventriculoperitoneal (VP) shunt involves diversion of cerebrospinal fluid (CSF) from the ventricular system to the peritoneal cavity at a rate determined by the characteristics of the valve device. When the rate at which the CSF enters the cavity is higher than its capacity to absorb it is overwhelmed, ascitis can occur, with abdominal distension and increase in intraperitoneal pressure.

A number of abdominal complications have been reported following the VP shunt placement.[1–5] Burst abdomen is however rare. Our review of the literature on abdominal complications following shunt insertion did not reveal any reported case of burst abdomen following the procedure. In this paper, we report a case of burst abdomen following shunt insertion in a 14-week-old baby. We believe that one major reason for the complication in this patient was the level of malnutrition in the face of other already existing risk factors.

Our patient is a 12-week-old baby who was admitted with progressive increase in head size, weight loss and vomiting. He had previously been admitted for a febrile illness and jaundice. The mother confirmed having had fever at 24 weeks of gestation.

Examination revealed an irritable, ill-looking and severely malnourished infant with edema of both hands and feet. His head circumference was 53.5 cm. The anterior fontanel was bulging and tense and his scalp was thin and shiny with engorged veins. There was global hypertonia and sunset phenomenon was evident. Investigations performed include skull X-ray, transfontanel ultrasound scan, full blood count, serum proteins, urea and electrolytes. The main positive findings were anemia, hypoproteinemia and electrolyte imbalance.

Because he was not fit for immediate surgical intervention, the patient was built up over the next 2 weeks. Treatment during this period included blood transfusion, intravenous fluids, correction of his electrolytes, vitamin supplements and increased breast feeding.

At the end of 2 weeks, with the normalization of his serum proteins, electrolytes and hematocrit, it was determined that he was relatively fit for surgery.

VP shunt was performed by standard methods using a medium pressure valve device.

The first few days after surgery were marked by increased bowel movements. By the sixth day after operation, the abdomen was observed to be distended and the abdominal wound dressing showed a slight bulge. On wound exposure, a loop of small bowel was seen protruding through the incision. The child was immediately transferred to our pediatric surgery unit and scheduled for emergency laparotomy.

At this operation, it was discovered that there was no evidence of commencement of wound healing and all the sutures applied to the abdominal wound at the time of the VP shunt had given way. Most of the distal small bowel was gangrenous. The affected segment, together with the ileo-cecal valve, was resected and jejuno-colic anastomosis was performed. Examination of the scalp wound also showed no evidence of healing. All the sutures had eroded through the tissues and had fallen off with exposure of the shunt device.

The postoperative course was turbulent. The patient remained hypertonic and had persistent electrolyte imbalance, anemia and sepsis. Attempts were made to correct these, but the patient continued to deteriorate and finally died 1 week later.

Burst abdomen is characterized by surgical wound disruption following abdominal operations, with herniation of the abdominal contents. Major predisposing causes include raised intra-abdominal pressure (as occurs in chronic obstructive uropathy, constipation, global hypertonia and chronic cough) and abdominal distension. Others include infections, hypoproteinemia and anemia. The latter causes delayed or poor wound healing. Abdominal complications following shunt surgery are not uncommon.[1,2] Well-known ones include peritonitis, volvulus, intestinal obstruction, ascitis, paralytic ileus, spontaneous extrusion of the silicone tube, abdominal cysts,[4,5] etc. Failure to thrive, occurring as a result of prolonged malnutrition, is a well-know feature of advanced hydrocephalus.[6] Essentially, it is the outcome of a combination of repeated vomiting and poor feeding found in many of these patients.

Our patient was markedly malnourished before surgery, and even though this was intensively treated as part of the preoperative preparation, it was obviously not enough to ensure adequate wound healing in the interval of 2 weeks.

In addition, the gross abdominal distension that occurred following diversion of fluid to the peritoneal cavity on the one hand and the global hypertonia with sustained raised intra-abdominal pressure on the other resulted in the sutures giving away easily. In malnourished patients with advanced hydrocephalus scheduled for shunting procedures, prevention of this complication must include measures aimed at ensuring that the nutritional status is adequate so as to facilitate wound healing. The operation might need to be delayed for as long as feasible. In the interim, other measures to control the intracranial pressure, such as external ventricular drainage, might be helpful.

Furthermore, the peritoneal perforation should be as tiny as possible and should be closed with nonabsorbable purse-string sutures. Where the stab wound is bigger, however, meticulous repair with narrow interval between stitches is recommended.
